# Agro‐morphological and nutritional assessment of chenopod and quinoa germplasm—Highly adaptable potential crops

**DOI:** 10.1002/fsn3.3502

**Published:** 2023-07-12

**Authors:** Rakesh Bhardwaj, Rashmi Yadav, Harinder Vishwakarma, Kriti Sharma, Rahul Chandora, Jai Chand Rana, Amritbir Riar

**Affiliations:** ^1^ ICAR– National Bureau of Plant Genetic Resources New Delhi India; ^2^ Alliance of Bioversity International and CIAT New Delhi India; ^3^ Department of International Cooperation Research Institute of Organic Agriculture FiBL Frick Switzerland

**Keywords:** dietary diversity, genetic diversity, human health, nutritional composition, quinoa

## Abstract

Quinoa belongs to the family *Chenopodiaceae*, a pseudo‐grain having high nutritional value and is considered an underexploited vegetable crop with the potential to improve the nutritional security of millions. Therefore, assessing genetic diversity in *Chenopodium* germplasm to untap nutritional and site‐specific adaptation potential would be of prime importance for breeders/researchers. The present study used 10 accessions of two *Chenopodium* species, that is, *C. quinoa* and *C. album*. Quantitative and qualitative phenotypic traits, proximate composition, minerals, and amino acids profiles were studied to compare the differences in nutritional value and extent of genetic diversity between these two species. Our results showed significant variation existed in yield attributing agro‐morphological traits. All the traits were considered for hierarchical clustering and principal components analysis. Large genetic variability was observed in traits of *Chenopodium* accessions. The protein, dietary fiber, oil, and sugar content ranged from 16.6% to 19.7%, 16.8% to 26%, 3.54% to 8.46%, and 3.74% to 5.64%, respectively. The results showed that *C. album* and *C. quinoa* seeds had good nutritional value and health‐promoting benefits. The *C. quinoa* was slightly ahead of than *C. album* in terms of nutritional value, but *C. album* accession IC415477 was at par for higher test weight, seed yield (117.02 g/plant), and other nutritional parameters with *C. quinoa* accessions. IC415477 and other potential accessions observed in this study may be taken up by breeders/researchers in the near future to dissect nutritional value of *Chenopodium* and related species for dietary diversity, which is imperative for the nutritional security of the ever‐growing world's population.

## Graphical abstract

Assessing genetic diversity in Chenopodium germplasm to untap nutritional and site‐specific adaptation potential would be of prime importance for breeders/researchers. The present study used 10 accessions of two Chenopodium species, that is, *C. quinoa* and *C. album*. Quantitative and qualitative phenotypic traits, proximate composition, minerals, and amino acids profiles were studied to compare the differences in nutritional value and extent of genetic diversity between these two species. Our results showed significant variation existed in yield attributing agro‐morphological traits. Potential accessions observed in this study may be taken up by breeders/researchers in the near future to dissect nutritional value of Chenopodium and related species for dietary diversity which is imperative for the nutritional security of the ever‐growing world's population.

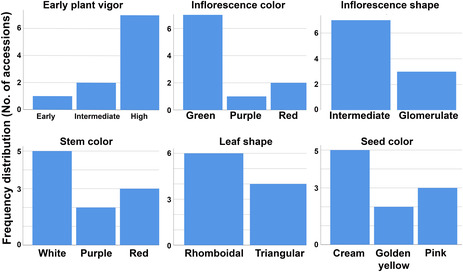

## 1 INTRODUCTION

Modern food production is limited as the knowledge is restricted to a few hundred out of many thousand known food plants globally. Surveys based on ethno‐botanic studies indicated thousands of traditional species are ignored by food companies and scientific researchers. Moreover, increasing global demand for 3Fs, that is, food, fuel, and feed, has shifted the attention from cereals to pseudocereal crops as food production based on cereals is limiting nowadays. Pseudocereals are reported to have wider environmental stability and more potential to resist abiotic stresses. *Chenopodium* is a native species of Andes (> 5000 years) and has gained attention due to its good water use efficiency, tolerance to low soil moisture (less than 200 mm of annual rainfall), and wide adaptation to different climates, such as salinity, drought, frost, and acidity (Bazile, Jacobsen, & Verniau, 2016). People called *Chenopodium* a “Super cereal” due to its high protein content, oil content (1.8%–9.5%), balanced amino acid percentage (high lysine (5.1%–6.4%), and methionine (0.4%–1.0%)). It possesses high polyunsaturated fatty acids (PUFA), mainly oleate, linoleate, linolenate, natural antioxidants, and high minerals, particularly calcium, phosphorus, iron, magnesium, and vitamin content as compared to other cereals (Koziol, 1992; Repo–Carrasco–Valencia et al., 2010). *Chenopodium* spp. has been cultivated as a leafy vegetable and subsidiary grain crop for many years. Two species of Chenopodium (*C. quinoa* and *C. album*) are reported to have good nutritional value but most studies have mainly concentrated on *C. album* (Hussain et al., 2020). C*henopodium album*, commonly known as Chenopod, belongs to Leiosperma, whereas *Chenopodium quinoa* is recognized as Quinoa, and belongs to the Cellulata subsection. Chenopod and quinoa are considered pseudocereals because of their grain characteristics similar to other cereal crops. They are recognized as potential crops owing to their nutritional value and ability to survive under adverse soil and climate conditions (Aguilar & Jacobsen, 2003; Fuentes & Bhargava, 2011). Some antinutritional substances have also been reported in Chenopodium, such as saponins, phytic acid, tannins, and protease inhibitors (Vega–Gálvez et al., 2010). Moreover, based on reports by clinicians, the plant has potential ingredients to counter breast cancer (Khoobchandani et al., 2009). Overall, Chenopodium is a potential crop that would contribute to healthier diets and nutritional security in various world regions with great nutritional value because of its high protein content, essential amino acids, vitamins, minerals, carbohydrates, isoflavones, and unsaturated fats (Jaikishun et al., 2019; Pinto et al., 2020; Sampaio et al., 2020). The basic chromosome number (X) for *Chenopodium* species is 9, that is, 2*n* = 4*x* = 36. *C. album* exists as diploid, tetraploid, and hexaploid, while *C. quinoa* is generally tetraploid (Maughan et al., 2006). Chenopod is widely distributed on all continents; although considered an annual weed in many places, extensively cultivated as a food crop in Northern India and is locally known as Bathu or Bathwa. In India, Chenopod is represented by about 21 species, some cultivated as vegetables and a few for grains (Yadav et al., 2007). The average grain yield is reported to be 0.25–0.47 t ha^−1^. A tall, robust form previously regarded as *C. giganticum* is now recognized as a variety of *C. album* L. Steward, cultivated in the Northwestern hills of India. The breeding program to improve Chenopodium is restricted because of lacking genetics and genomics information. Therefore, knowledge of genomic variation, genetic diversity, and population structure is necessary (Zhang et al., 2017). That is why the evaluation of plant material is necessary for any breeding program.

In developing countries such as Africa, Chenopodium has been introduced into routine agricultural systems to fulfill the purpose of regional food security (Babatunde et al., 2011; Bazile, Pulvento, et al., 2016). Phenotypic plasticity, wide variability, and agronomic adaptation make Chenopodium survive across hot arid to subtropical humid climates. However, it is necessary to select and introduce different Chenopodium genotypes for a wider range of environments. Moreover, domestication has narrowed down the genetic variability of *Chenopodium*, so it is essential to explore genetic variability that still exists for seed color, leaf shape, leaf color, grain yield, and resistance against biotic and abiotic stresses to explore the potential of Chenopodium for nutrition security and climatic adaptability. *Chenopodium album* is native to India, with huge germplasm diversity available, while *C. quinoa* is an exotic species. Chenopod and quinoa grain flour is gluten‐free and has rheological properties, sensory characteristics, and texture similar to corn flour. The consumption of Quinoa has increased substantially worldwide, whereas Chenopod is not exploited for its potential, owing to the limited information available on its nutritional value. Previous studies have estimated agro‐morphological parameters of cultivated materials, evaluated physiological parameters in different quinoa varieties at vegetative and reproductive phases, and established a taxonomic key for identifying types (Infante et al., 2018). Previously authors studied the intrapopulation phenotypic variation in Piartal material and found phenotypic variation in Chenopodium species (Manjarres‐Hernández et al., 2021; Morillo et al., 2020). Along with morphological markers, researchers have used molecular markers such as SSR markers, and SRAP markers for the characterization of Chenopodium genotypes (El‐Harty et al., 2021). Moreover, the pangenome of 21 Chenopodium accessions has revealed information about important genes that play an important role in abiotic stress tolerance against adverse environmental conditions (Schmockel et al., 2017). Previous studies related to Chenopodium were mostly carried out in growth chambers under controlled conditions, but evaluation of agro‐morphological traits under field conditions is rare. Therefore, critical functional traits should be studied under field conditions before introducing in a particular environment.

To fill this gap, we have studied morphological and nutritional characteristics of 10 *Chenopodium* accessions in two species (*C. quinoa* and *C. album*) under field conditions to identify the degree of similarity/dissimilarity among germplasm lines based on agro‐morphological traits and nutritional traits. So, an attempt was made to compare nutritional value and genetic divergence of agro‐morphological traits in two *Chenopodium* species. Additionally, identifying Chenopodium accession based on agro‐morphological and nutritional traits would helps in selecting better genotypes for farmers, breeders, researchers, and marketers.

## 2 MATERIALS AND METHODS

### 2.1 Plant material

Five accessions of each *C. quinoa* and *C. album* were collected from the National Bureau of Plant Genetic Resources, Regional Station, Shimla, Himachal Pradesh, India. The seeds were grown in 10 replicates, each under field conditions for 2 consecutive years (2015–16 and 2016–17), and appropriate agronomic practices were followed. The plants were grown to maturity, and essential parameters were recorded at an appropriate time.

### 2.2 Variability in agro‐morphological traits of Chenopodium accessions

The data were recorded on 10 plants for each accession for 18 morphological traits (quantitative). These traits were early plant vigor, growth habit, inflorescence length and color, stem color, leaf color, leaf shape, seed color, days to 50% flowering, leaf length and width, plant height, days to 80% maturity, seed yield per plant, and test weight (weight of 1000 seeds in g). The descriptor issued by Bioversity International was followed for data recording of *Chenopodium* accessions (Bioversity International, FAO, PROINPA, INIAF, and IFAD, 2013).

### 2.3 Estimation of total protein, soluble starch, phytate content, amino acids, and mineral profiling of Chenopodium accessions

Seeds from mature crops were taken for nutritional profiling. The seed color was recorded before proceeding with further steps. Seeds were dried in an oven at 60°C overnight, cooled to room temperature in a desiccator, ground in a cyclone mill, and passed through a 0.5 mm Tyler sieve for further nutrition component analysis. Proximate composition of *Chenopodium* samples was carried out as per AOAC (Association of Official Analytical Chemists) methods, namely moisture content (AOAC 934.01), ash content (AOAC 938.08), fat (AOAC 920.58), protein content (AOAC 985.29) and dietary fiber (AOAC 985.29) (AOAC, 1994). Total soluble sugar content was estimated by phenol–sulfuric acid method (Dubios et al., 1956). Starch content in *Chenopodium* accessions was estimated as per AOAC 996.11. Total phenol content was estimated as per previously standardized protocol (Singleton & Orthofer, 1999). Enzymatic–colorimetric method was used for estimating phytate content in *Chenopodium* accessions. Phytate was hydrolyzed by enzyme phytase and alkaline phosphatase, and released phosphorous was estimated as per K–Phyt (phytic acid assay kit (Megazyme, Ireland)). Mineral (Cu, Fe, Zn, and Mg) profiling was done as per AOAC 985.35. ASFRM‐6 (fish meal) and ASFRM‐14 (rice flour) were used as reference standards for method validation. Amino acids profiling was done using waters AccQ.Tag™ method as reported (Manivannan et al., 2018).

### 2.4 Statistical analysis of Chenopodium accessions under study

The data were taken for 2 consecutive years, and the mean, minimum, maximum, and standard deviation were calculated for each accession. The dissimilarity matrix was used to construct a dendrogram by the unweighted pair group method for the arithmetic mean (UPGMA)‐based Sequential Agglomerative Hierarchical and Nested (SAHN) clustering. Multivariate statistical methods have been successfully employed to classify qualitative and quantitative traits in many crop plants, but such studies are lacking in Chenopodium (Vasconcelos et al., 2016). PCA and cluster analysis tools were used to assess accessions and how closely they are related to each other. Information on interrelationships among traits is a powerful tool for designing appropriate breeding strategies. Principal component analysis (PCA) based on the Euclidean coefficients of dissimilarity was done. All these calculations were done using NTSYS‐pc version 2.11 (USA) (Rohlf, 2000). Pearson's correlation (average inter‐ and intracluster D^2^ values) between qualitative and quantitative traits of *Chenopodium* was carried out using SPSS software. Biplot analysis was carried out using Minitab software (https://www.minitab.com/en‐us/).

## 3 RESULTS

### 3.1 Variability in agro‐morphological traits of Chenopodium

Analysis of variance in 10 accessions for two *Chenopodium* species showed differences in all 18 traits. The quantitative traits of both *Chenopodium* species were measured (Table 1) and observed that *C. quinoa* started flowering early, days to 50% flowering ranged 37–61 days (IC411824 & IC411825), whereas in *C. album*, it ranged from 65 to 107 days. We found that *C. quinoa* plants reached 80% maturity in 78–139 days compared to *C. album*, which showed maturity in 110–161 days. Interestingly, *C. album* had a longer inflorescence length (40.2–57.2 cm) (accession IC258253) as compared to *C. quinoa* (26.2–35.7 cm). We have also observed that *C. album* (152.5–366.4 cm) plants were taller and had long and wide leaves compared to *C. quinoa* (109–127.2 cm). The seed yield per plant ranged from 55.40 to 62.16 g in *C. quinoa*, while in *C. album*, it was 54.36 to 117.02 g. Seeds of *C. quinoa* were bold, having test weight (weight of 1000 seeds) in the range 1.05–1.35 g (EC507741) as compared to *C. album* seeds (0.4–1.3 g) (Table 1). The general statistical parameters for all the traits in respect of 10 accessions (Tables 1 and 2) showed a wide range and high variance for different traits included in the study. The coefficient of variance (%) showed the presence of the highest variation for the traits, namely 1000 seed weight, beak length, leaf petiole length, and leaf width as compared to other traits. Seed yield (54.36–117.02 g/plant), oil percent (27.7%–43.2%), days to 50% flowering (37–103 days), and the number of siliques/plant (2.3–73.2 g) showed a wide range of phenotypic expression in the collection. The wide range and high coefficient of variation for different characters observed under study indicated the presence of good variability in the collection.

**TABLE 1:** Estimation of *Chenopodium quinoa and C. album* for different agro‐morphological traits.

		Qualitative traits								Quantitative traits							
Accessions	Species	Early plant vigor	Plant growth habit	Infl. color	Infl. shape	Stem color	leaf color	leaf shape	Seed color	Days to 50% flowering	Infl. length (cm)	leaf length (cm)	Leaf width (cm)	Plant height (cm)	Days to 80% maturity	Seed yield/plant (g)	1000 Seed wt (g)
IC108808	*C. album*	3	Simple	Red	Intermediate	White	Green	Rhomboidal	Pink	103 ± 2.51	51.85 ± 2.11	10.8 ± 0.67	8.7 ± 0.65	357.4 ± 12.34	147 ± 4.33	85.08 ± 2.33	0.70 ± 0.03
IC109734		3	Simple	Green	Glomerulate	Purple	Green	Rhomboidal	Pink	107 ± 3.82	40.25 ± 2.18	12.8 ± 0.65	13.7 ± 1.23	366.4 ± 10.56	161 ± 4.55	86.61 ± 3.33	0.80 ± 0.03
IC109733		3	Simple	Purple	Intermediate	Red	Green	Rhomboidal	Golden yellow	77 ± 3.51	53.60 ± 3.23	10.2 ± 0.78	5.2 ± 0.78	265.8 ± 11.45	145 ± 3.4 s	79.73 ± 3.56	1.00 ± 0.04
IC258253		1	Simple	Green	Intermediate	Purple	Green	Triangular	Golden yellow	70 ± 5.29	57.20 ± 2.33	6.5 ± 0.65	2.0 ± 0.34	152.5 ± 15.66	129 ± 3.56	54.36 ± 3.67	0.40 ± 0.02
IC415477		3	Simple	Green	Glomerulate	White	Green	Rhomboidal	Pink	65 ± 2.08	46.65 ± 3.32	15.3 ± 1.11	11.1 ± 0.98	218.2 ± 16.45	110 ± 6.78	117.02 ± 5.44	1.30 ± 0.04
EC507741	*C. quinoa*	3	Simple	Green	Glomerulate	White	Green	Triangular	Cream	59 ± 4.72	28.20 ± 1.23	4.6 ± 0.54	2.3 ± 0.08	109.0 ± 7.89	102 ± 5.67	57.64 ± 3.45	1.35 ± 0.03
EC507742		3	Simple	Red	Glomerulate	White	Green	Triangular	Cream	61 ± 2.11	35.70 ± 1.98	3.8 ± 0.98	1.9 ± 0.04	111.2 ± 10.67	110 ± 5.67	62.16 ± 4.45	1.12 ± 0.04
EC507744		3	Simple	Green	Glomerulate	White	Green	Triangular	Cream	58 ± 2.08	29.00 ± 1.23	3.7 ± 0.76	1.6 ± 0.06	106.2 ± 6.78	102 ± 7.78	59.68 ± 4.23	1.05 ± 0.03
IC411824		2	Simple	Green	Glomerulate	Red	Green	Rhomboidal	Cream	37 ± 3.64	26.20 ± .98	8.0 ± 0.62	5.2 ± 0.68	127.2 ± 5.89	78 ± 4.56	55.40 ± 3.45	1.20 ± 0.04
IC411825		2	Simple	Green	Glomerulate	Red	Green	Rhomboidal	Cream	37 ± 2.08	29.10 ± 1.12	8.1 ± 0.55	5.5 ± 0.68	121.7 ± 11.34	139 ± 8.77	59.03 ± 4.56	1.15 ± 0.03

*Note*: Early plant vigor: 1—Low, 2—Intermediate, and 3—High.

**TABLE 2:** Proximate, phytochemical, and mineral content in different accessions of *C. album* and *C. quinoa.*

Sps.	Accession No.	Moisture	Ash	Protein	Dietary fiber	Oil	Sugar	Starch	Phenol	Saponin	Cu	Fe	Zn	Mg
		%									mg/100 g			
*Chenopod quinoa*	IC411824	11.2^cde^	3.12^cd^	17.2^ab^	17.9^a^	3.79^a^	5.64^b^	44.6^c^	0.441^a^	0.187 ^abcd^	1.50^ef^	23.4^c^	8.94^de^	214^c^
	IC411825	11.7^e^	2.12^a^	18.2 ^bc^	17.2 ^a^	3.54^a^	5.34^ab^	45.3^d^	0.429 ^a^	0.137^a^	1.60^f^	22.8^c^	10.7^f^	198^bc^
	EC507741	11.4^de^	3.17^cd^	19.7^d^	21.7^b^	3.67^a^	4.56^ab^	36.9^b^	0.539^bc^	0.153^abc^	1.91^g^	31.7^e^	8.44^cde^	242^d^
	EC507742	11.3 ^cde^	2.54^ab^	19.0^cd^	16.8 ^a^	4.67^ab^	4.37^ab^	44.4^bc^	0.533^bc^	0.144 ^ab^	1.31^cd^	24.6^cd^	7.81^c^	192^bc^
	EC507744	10.5^ab^	3.24^d^	18.1 ^bc^	20.9^b^	5.22^b^	4.80^ab^	38.8 ^bc^	0.499^b^	0.179 ^abcd^	1.51^ef^	26.6^dc^	8.28^cd^	269^e^
	Mean	11.2	2.84	18.4	18.4	4.18	4.94	41.9	0.486	0.160	1.57	25.8	8.83	223
*Chenopod album*	IC108808	11.4 ^de^	3.20^d^	18.5^c^	25.8^c^	6.64^c^	3.74^a^	32.7^a^	0.692^d^	0.158 ^abc^	1.26^bcd^	12.9^ab^	6.99^b^	198^bc^
	IC109734	10.8^bc^	2.83^bc^	18.3 ^bc^	23.9 ^c^	7.72^cd^	3.83 ^a^	33.3 ^a^	0.578^c^	0.203^cd^	1.04^a^	13.4^ab^	5.30^a^	183^b^
	IC109733	11.0^bcd^	2.51^ab^	16.6^a^	25.3 ^c^	6.86^c^	3.97 ^a^	32.5 ^a^	0.732^d^	0.224^d^	1.18^bc^	11.7^a^	6.39^b^	267^e^
	IC258253	10.0^a^	2.66^bc^	17.3 ^ab^	17.7 ^a^	8.46^d^	4.55^ab^	44.6^c^	0.549^bc^	0.220^d^	1.38^de^	35.8^f^	7.91^c^	243^d^
	IC415477	10.5^ab^	2.35^ab^	18.2^bc^	26.0^c^	7.82^cd^	4.08^ab^	32.7 ^a^	0.693^d^	0.190^bcd^	1.12^ab^	14.4^b^	9.24^e^	157^a^
	Mean	10.7	2.71	17.9	23.4	7.50	4.04	33.2	0.651	0.199	1.20	17.7	7.16	210

*Note*: Superscript letters represent significant variation with *p* ≤ .05.

**FIGURE 1:**
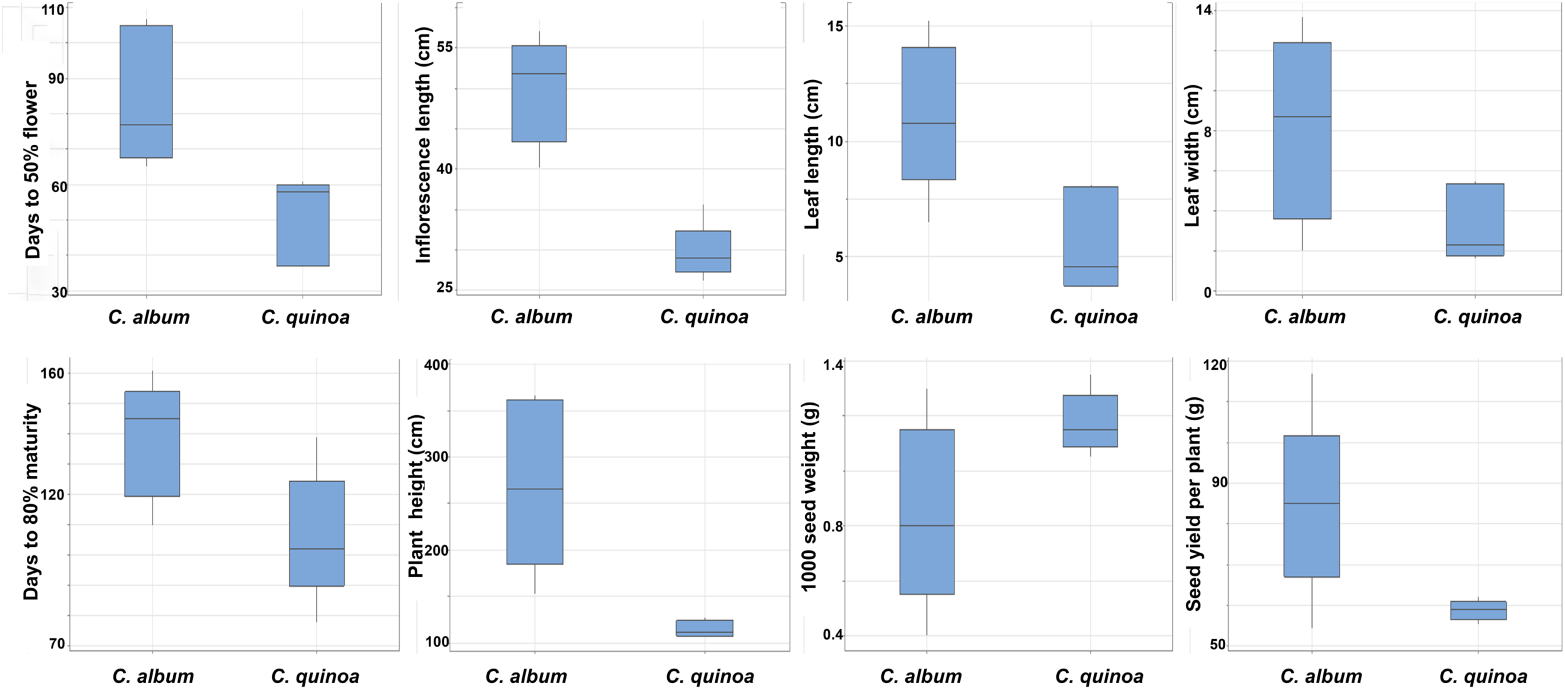
Quantitative traits in *Chenopodium* species represented by box plot.

**FIGURE 2:**
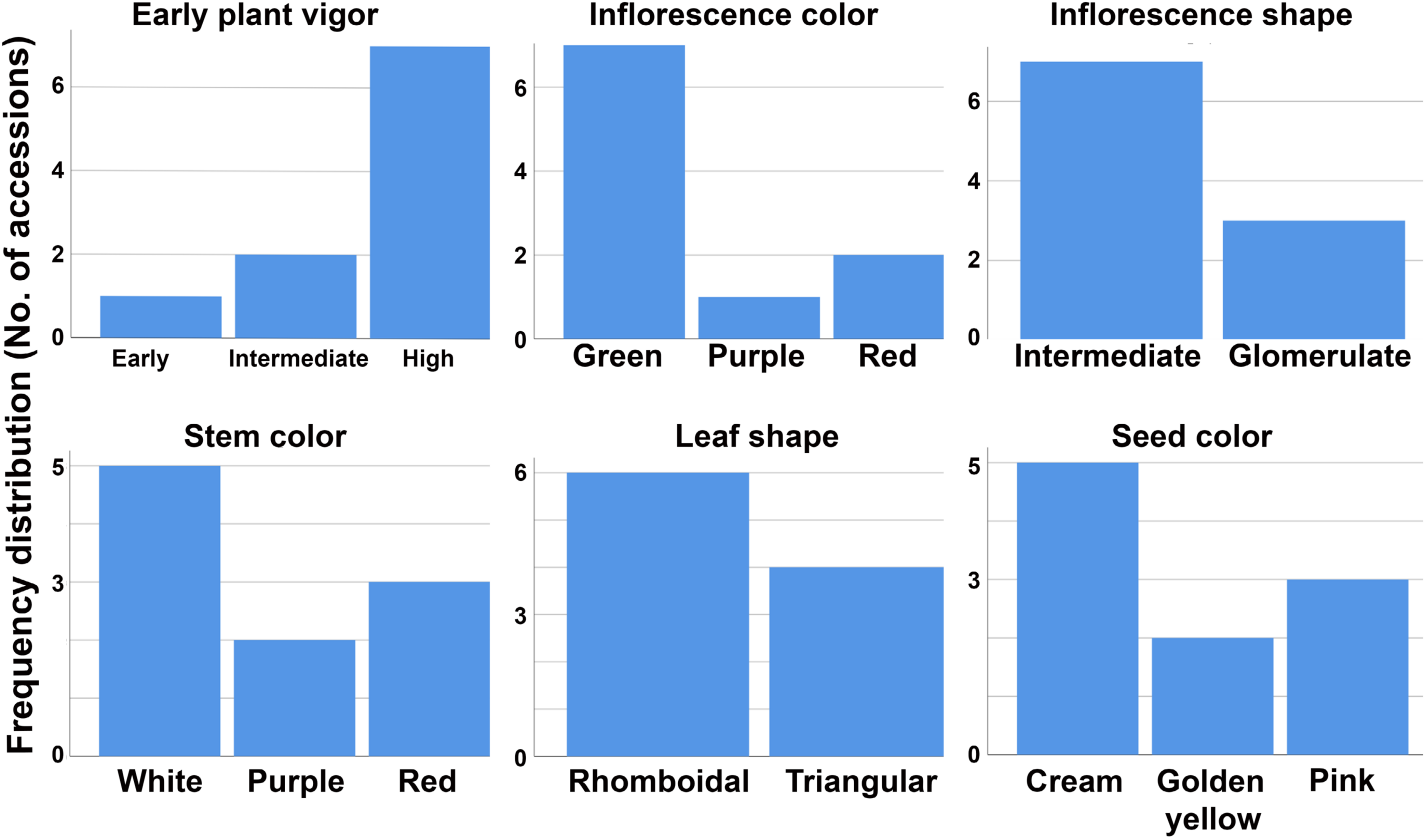
Frequency distribution of qualitative traits in *Chenopodium* species.

All the agro‐morphological traits were comparatively high in Chenopodium album compared to *C. quinoa* except for the test weight (weight of 1000 seeds) (Figure 1). Box plot representation indicated significant variation among the different accessions regarding most of the characters investigated, indicating sufficient variability in the material used for the study (Figure 1). Among qualitative traits, high plant vigor was observed in most of the accessions (7 of 10). The accessions of both *C. album* and *C. quinoa* showed simple growth habit. Green inflorescence color was dominant in both *C. album* and *C. quinoa* followed by red color inflorescence. Accession IC109733 was observed to have inflorescence of purple color. All *C. quinoa* accessions possessed glomerulate type inflorescence shape while *C. album* accession showed diversity in inflorescence shape both possessing intermediate and glomerulate type. Green leaf color was observed in all the accessions of *C. quinoa* and *C. album*. Single seed color (cream) was present in all *C. quinoa* accessions while *C. album* showed diverse seed colors with pink and golden yellow seeds (Table 1; Figure 2). Systematic efforts were to analyze variability among different germplasm existing in India for traits, namely days to 50% flowering, days to 80% maturity, plant height, number of capitulating per plant, seed yield, oil content, and protein content.

### 3.2 Proximate composition analysis of Chenopodium accessions

Mean composition values revealed that *C. quinoa* has a slightly higher content of moisture, ash, protein, and total sugar and a much higher content of starch than *C. album* (Table 2). In contrast, dietary fiber and oil content were much higher in *C. album*. In *C. quinoa*, accession EC507744 has the lowest moisture content (10.5%), close to the mean value (10.7%). Ash content in *C. quinoa* ranged from 2.1 to 3.24%, accession EC507744 (3.24%) had the highest ash content followed by EC507741 (3.17%) and EC507744 (3.120%), while in *C. album*, the ash content was highest in IC108808 (3.20%) followed by IC109734 (2.83%). *C. quinoa* accession EC507741 had the highest protein content of 19.7%, while the highest protein content in *C. album* was 18.5% in accession IC108808. Our results showed that dietary fiber ranged from 16.8%–21.7% in *C. quinoa* and 17.7%–26% in *C. album*, indicating that C. quinoa has a lower dietary fiber content than *C. album*. But *C. quinoa* accessions EC507741 and EC507744 had 21.7% and 20.9% dietary fiber, respectively, which is closer to the mean value of *C. album* (23.4%).

In Quinoa, the highest oil content was 5.22% (EC507744), which was lower than the lowest value of *C. album* accession IC108808 (6.64%). So, high variability was observed in *Chenopodium* accessions for oil content trait. Total soluble sugars content was at par in both species, and differences were only marginal, as reflected in DMRT (Duncan's multiple‐range test) grouping. However, the starch content of the *C. quinoa* group (41.9%) was far higher than the *C. album* group (33.2%). *C. quinoa* accessions EC507741 and EC507744 had the lowest starch content in the group 36.9% and 38.8%, respectively.

### 3.3 Phytochemical content analysis of Chenopodium accessions

Total phenols and total steroidal saponins were estimated, and it was revealed that the *C. album* group had a much higher content of phenols and saponins (Table 2). However, quinoa accessions EC507741 and EC507442 have higher phenol content (0.539% and 0.533%, respectively), which was equivalent to the phenol content of *C. album* group accessions IC109734 and IC258253 (0.578% and 0.549%, respectively). The highest saponin content in the quinoa group was 0.187% (IC411824) and 0.179% (EC507744), which was close to the mean value of the *C. album* group (0.199%), while the lowest saponin content of *C. album* group was 0.158% (IC108808), which was close to mean value of *C. quinoa* group (0.160%).

### 3.4 Mineral content analysis of Chenopodium accessions

Ash content is the sum of total minerals; here, we observed that *C. quinoa* had a much higher content of ash as compared to *C. album* (Table 2). We may also conclude that individual minerals were higher in *C. quinoa*. The mineral contents were present in the following order Cu < Zn < Fe < Mg, in both *Chenopodium* species. However, the distribution of individual minerals may vary. The marginal differences were observed between the mean values of both groups for copper, zinc, and magnesium (1.06 to 1.3 times). In contrast, the mean iron content of the quinoa group was 1.46 times higher than the iron content of *C. album* group. Our results revealed high genetic variability in the *C. quinoa* and *C. album* accessions. The maximum zinc content in *C. quinoa* was recorded in accession IC411825 and the minimum in EC507742. Maximum ash and magnesium content were recorded in accession EC507744 and minimum in EC507742 and IC411825. Maximum protein, copper, and iron content were recorded in accessions EC507741 and EC507744 and minimum in IC411824 and EC507742. The maximum content of oil and iron in the *C. album* was recorded in accession IC258253 and the minimum in IC108808. The maximum zinc and magnesium content was recorded in accessions IC415477 and IC109733, whereas the minimum was in IC109734. The highest iron content was found in *C. album* accession IC258253 (35.8 mg/100 g), significantly more than the highest iron‐containing quinoa accession EC507741 (31.7 mg/100 g). The highest zinc content in the quinoa group was 10.7 mg/100 g (IC411825), whereas, in the *C. album* group, it was 9.24 mg/100 g (IC415477).

### 3.5 Amino acids profiling in Chenopodium accessions

**FIGURE 3:**
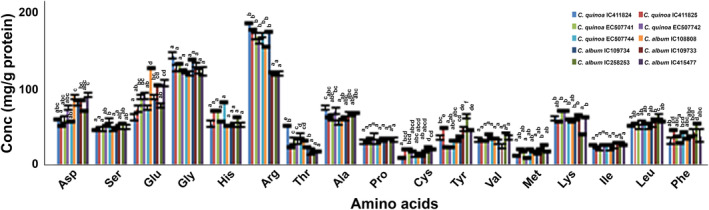
Variability in amino acids concentration of *Chenopodium quinoa* and *C. album*. Different superscript letters on top of the error bar represent differences in significance.

Amino acid profiling of *C. quinoa* and *C. album* showed nonsignificant differences for glycine, histidine, proline, valine, and isoleucine as values for these amino acids formed a single group in DMRT. Serine, methionine, leucine, and lysine showed significant intra‐/intergroup variability (Figure 3). The high serine content of quinoa accessions (EC507742 and EC507744) was at par with *C. album* accessions (IC109733, IC258253, and IC415477). Three accessions of *C. quinoa* and four accessions of *C. album* were statistically at par, having high methionine content. The highest protein content (28.5 mg/g) was observed in *C. album* accession IC258253. Similarly, the amino acid leucine was also high in *C. album* accession IC258253 (protein content 66.2 mg/g). The mean lysine content (65.9 mg/g protein) in Quinoa was higher than *C. album* (58.6 mg/g protein), with the highest value of 73.5 mg/g protein in Quinoa (EC507741) and 66.6 mg/g protein in *C. album* (IC109733). In Quinoa, the mean value of arginine and threonine content (177 mg/g protein and 36.7 mg/g protein, respectively) was higher than the mean value in *C. album*. Two accessions of *C. album*, IC108808, and IC109734, had a high content of arginine and threonine, which were statistically at par with *C. quinoa* accessions. The mean value of aspartate, glutamate, tyrosine, phenylalanine, and cystine in *C. album* was higher than in Quinoa. However, the range of variability for these amino acids was similar in both groups. Accession IC415477 had the highest aspartate content (93.5 mg/g protein), IC108808 showed highest glutamate (130 mg/g protein), while accession IC258253 showed highest tyrosine (65.9 mg/g protein), phenylalanine (55.1 mg/g protein), and cystine (24.4 mg/g protein). All these accessions belonged to the *C. album* group (Table 3).

**TABLE 3:** Eigenvalue and percentage of variation explained by first five principal components and correlations between PC scores and agronomic traits of *Chenopodium* accessions.

Component matrix					
	Component				
	1	2	3	4	5
Moisture	−0.624	−0.463	−0.067	0.251	−0.383
Ash	−0.325	−0.279	0.369	−0.585	0.411
Protein	−0.426	−0.352	0.480	0.546	0.206
Oil	0.937	0.048	0.013	−0.106	0.101
Dietary fiber	0.566	−0.678	0.264	−0.177	−0.229
Sugar	−0.756	0.510	−0.315	−0.091	−0.142
Starch	−0.594	0.729	−0.259	0.148	0.125
Phenol	0.811	−0.462	0.184	0.006	−0.070
Saponin	0.696	0.237	−0.278	−0.565	0.075
Cu	−0.770	0.318	0.335	0.051	−0.106
Fe	−0.424	0.724	0.179	0.120	0.340
Zn	−0.522	0.440	−0.036	0.423	−0.376
Mg	−0.076	0.429	0.354	−0.574	0.124
Aspartic acid	0.814	−0.537	−0.089	0.087	0.080
Serine	0.281	0.534	0.455	−0.090	0.474
Glutamic acid	0.608	−0.615	0.318	0.239	−0.124
Glycine	−0.392	−0.180	−0.595	−0.430	−0.111
Histidine	−0.503	0.537	0.608	−0.055	0.000
Arginine	−0.865	−0.246	−0.045	−0.182	0.045
Threonine	−0.774	−0.222	−0.129	−0.369	0.253
Alanine	−0.064	0.058	−0.883	0.249	0.122
Proline	0.528	0.025	−0.148	0.429	0.501
Cystine	0.307	0.467	0.192	0.674	−0.259
Tyrosine	0.556	0.677	−0.246	−0.045	−0.380
Valine	0.090	0.419	−0.031	0.533	0.521
Methionine	0.355	0.734	0.333	−0.183	−0.383
Lysine	−0.366	−0.638	−0.015	0.197	0.151
Isoleucine	0.575	0.599	−0.397	−0.018	0.252
Leucine	0.617	0.749	−0.106	0.005	0.125
Phenylalanine	0.318	0.814	0.231	−0.167	−0.351
Eigenvalue	9.6	7.7	3.2	3.2	2.1
% Variation explained by Eigen root	32.0	25.6	10.9	10.4	7.3

### 3.6 Principal component analysis of Chenopodium accessions

**FIGURE 4:**
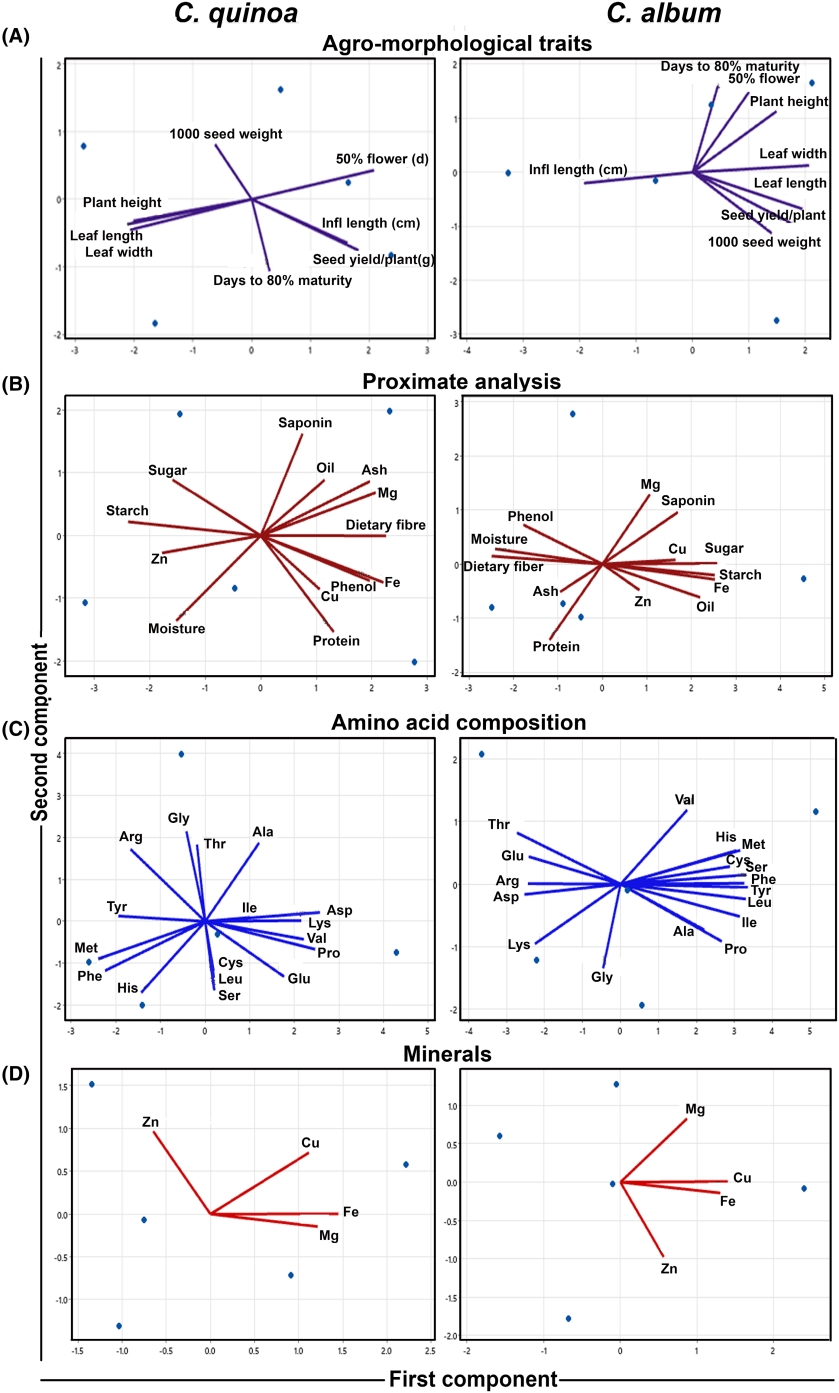
Biplot analysis of important traits in *Chenopodium* species. (a) Biplot analysis of agro‐morphological traits; (b) Biplot analysis of proximate composition; (c) Biplot analysis of amino acid contents; (d) Biplot analysis of mineral contents.

Principal component analysis of quantitative agro‐morphological traits in *C. quinoa* revealed days to 50% flowering, seed yield per plant, and inflorescence length were major contributors for PC1; both PC1 and PC2 contributed 83.8% genetic variability. For *C. album*, leaf length, leaf width, and seed yield per plant were major contributors for PC1 while days to 50% flowering and days to maturity were major contributors for PC2. Both PC1 and PC2 contributed 93% genetic variability (Figure 4a). PCA extracted a total of three components, among qualitative traits which accounted for 82.324% genetic variability. A majority of color‐related qualitative traits including seed color and inflorescence color contributed to overall variability as observed from the Eigen values. Based on hierarchical clustering, the qualitative traits were classified into three major clusters. Similarly, PCA of nutrient profiling in *C. quinoa* revealed dietary fiber, phenol, and ash content were major contributors to PC1, while moisture and protein content were major contributors to PC2. The first two PCs contributed a genetic variability of 81.2%. In case of *C. album*, the first two PCs contributed to 85.1% variability (Figure 4b). Amino acid profiling in *C. quinoa* revealed Asp, Valine, and Lys, as major contributors of PC1 while Gly, Ala, and Thr as major contributors of PC2. Both PC1 and PC2 contributed 72.1% genetic variability (Figure 4c). Amino acid profiling in *C. album* revealed Tyr, Phe, and Leu were major contributors of PC1 while amino acids Val, Thr, and Met were major contributors of PC2. Both PC1 and PC2 contributed 82.4% genetic variability. In relation to mineral contents, in the case of *C. quinoa*, PC1, and PC2 contributed to 87.5% genetic variability, while for *C. album*, PC1, and PC2 showed 86.7% genetic variability (Figure 4d).

### 3.7 Cluster analysis of Chenopodium accessions

**FIGURE 5:**
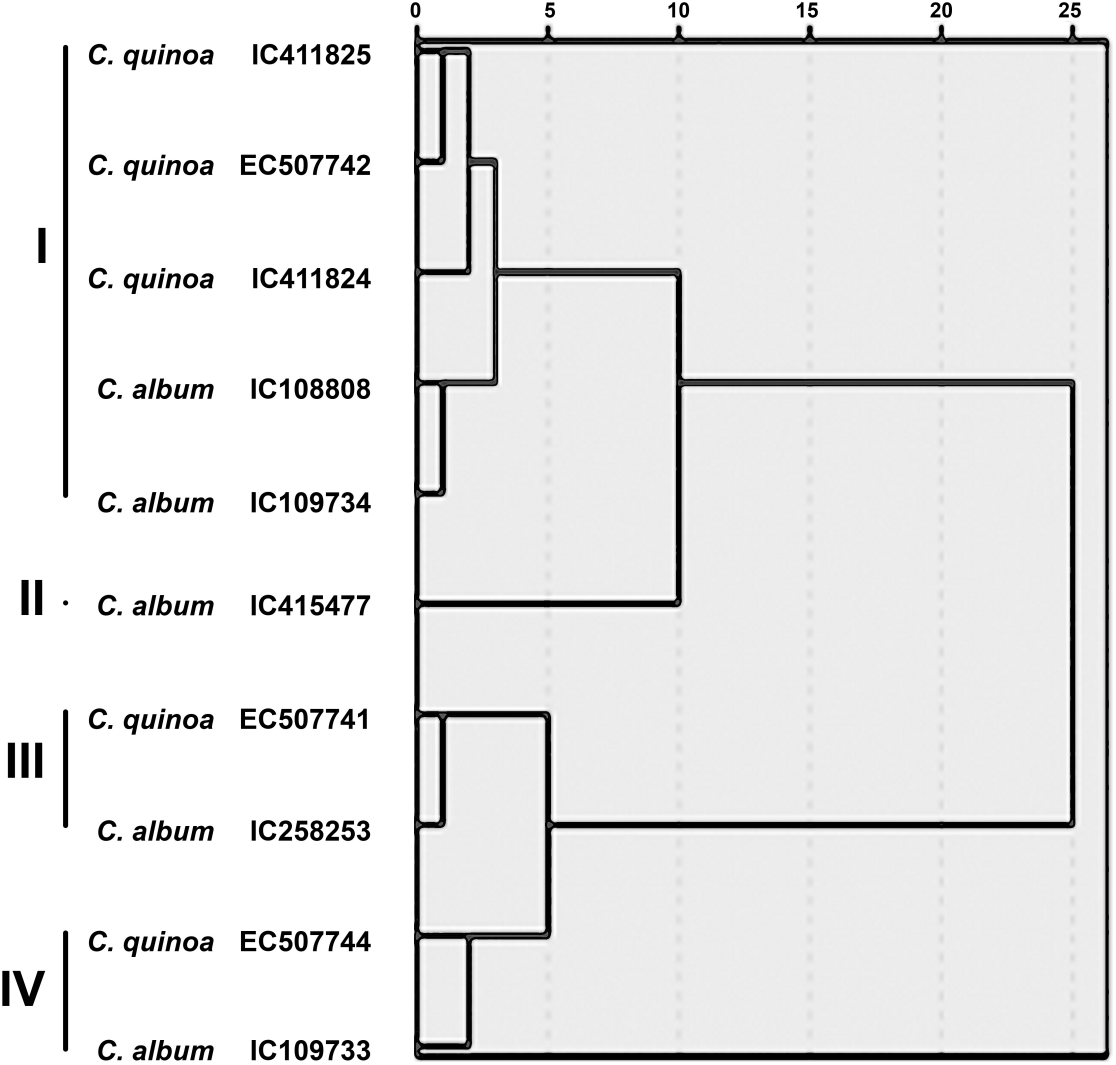
Dendrogram depicting close relatedness between different accessions of *Chenopodium* species.

The *Chenopodium* accessions were grouped into four clusters (at a standardized Euclidean distance of 5) based on the average linkage method (Figure 5). Clusters I and III consisted of five accessions, and clusters II and IV consisted of four lines each. Accessions from *C. quinoa* and *C. album* were distributed in various clusters, and no relationship was established between the origin and clustering pattern. The accessions in cluster I showed early maturity and high yield. Cluster II comprised lines with low seed quality but higher leaf quality components. Cluster III had the highest seed yield and high value for protein. The lines in cluster IV matured earliest and had high seed protein, while clusters II and IV had high seed yield, dry weight/plant, stem diameter, and maximum inflorescences. Cluster VI had low values for traits related to seed morphology and quality. There was considerable overlapping among clusters I, II, III, and IV, suggesting that principal components do not effectively separate the lines.

Accessions in the same cluster are biochemically similar, interestingly *C. quinoa* accession EC507741 and *C. album* accession IC258253 were present in the same cluster, Similarly, accessions EC507744 and IC109733 were present together in another cluster. Although they belong to different species and agroecological origins, they are very well adapted and closely associated with biochemical traits. On the other hand, quinoa accessions IC411825 and EC507744 were most distantly related, as observed in the hierarchical clustering. Thus, it indicates that in terms of phenotypic expression for biochemical traits, species boundaries are nonexistent, and during the evolutionary process, genes for nutritional traits have co‐inherited.

### 3.8 Correlation between traits of Chenopodium accessions

The correlation analysis between traits of *Chenopodium* accessions revealed that oil and saponin content were negatively correlated with moisture content. Protein content was negatively correlated with saponin (Table S1). Fifty percent flowering was positively correlated with plant height and maturity. The longer the inflorescence, the lesser the seed test weight. The probable reason behind that could be the ratio of source (inflorescence) to sink (seed) is getting affected as the inflorescence length increases to more than a certain extent. Leaf length was positively correlated with leaf width, plant height, and seed yield. Dietary fiber was negatively correlated with sugar, starch, iron content, and seed coat color. Dietary fiber was positively correlated with phenol content, 50% flowering, and yield per plant. Oil content correlated negatively with sugar, starch, copper, test weight, and seed coat color, while positively with saponin, yield per plant, and 50% flowering. Sugar content was positively correlated with starch, mineral content (Cu, Fe, and Zn), and seed coat color, while sugar content was negatively correlated with phenol content, time of maturity, yield per plant, and 50% flowering. Starch content was negatively correlated with phenol content, yield per plant, early plant vigor, and 50% flowering. Phenol content was positively correlated with 50% flowering and yield per plant, while it was negatively correlated with mineral content (Cu, Fe, and Zn) and seed coat color. Interesting results were obtained with the mineral content of *Chenopodium*. Copper was positively correlated with seed coat color and negatively correlated with yield per plant. Similarly, Fe was negatively correlated with yield per plant. Zn was negatively correlated with 50% flowering. The 80% maturity and 50% flowering were positively associated with each other (Table S1).

## 4 DISCUSSION

Our study indicated a large amount of genetic diversity existed in *Chenopodium* accessions. These variations are essential for developing new varieties with trait‐specific characteristics. We observed two different leaf shapes, that is, triangular (40%) and rhomboidal (60%), and the results were at par with the recent study (El‐Harty et al., 2021, 20). The panicle or inflorescence shape was glomerulate type in 70% of accessions and 30% of intermediate type. However, we did not observe an amarantiform type of panicle as observed recently (El‐Harty et al., 2021). The green color was dominating color in the leaf as well as in the panicle. Our results were in agreement with the recent study (El‐Harty et al., 2021). Bhargava and Ohri (2016) also observed various panicle, stem, and seed colors (Bhargava & Ohri, 2016). The average period of Chenopodium maturity slightly varies in different countries, for example, 109–163 days in India (Bhargava et al., 2007), 108–181 days in Denmark (Jacobsen, 2003), and 107–158 days in Turkey (Tan & Temel, 2018). Here, the accession matured at last was IC109734, which showed the highest plant height. It was in accordance with a recent study (El‐Harty et al., 2021). However, accessions maturing early have the advantage to escape the detrimental effect of heat stress at the end of the season. This genetic diversity is also reflected at the molecular level which is used by breeders globally to develop improved genotypes. Recent studies have used both morphological traits as well as molecular markers to characterize the Chenopodium (El‐Harty et al., 2021). Several previous studies have also used both molecular and phenotypic markers to investigate genetic diversity and population structure in Chenopodium (Christensen et al., 2007; Fuentes et al., 2009; Zhang et al., 2017).

Accessions with more genetic similarity were grouped during clustering analysis, and those with dissimilarity were placed far from each other in the dendrogram. The clustering pattern may be used in future research programs such as molecular assist breeding (MAB), conservation of species, genetics, and physiological studies. Previous studies based on *C. quinoa* and *C. berlandieri* suggested 6 clusters were formed using 31 lines (29 of *C. quinoa* and 2 of *C. berlandieri*), still 2 lines of *C. berlandieri* were placed at a distance in hierarchical clustering. The lines from same origin were not included in the same cluster which indicated heterogeneity within a geographical region. (Bhargava et al., 2007). Multivariate analysis showed that the first three PCs accounted for most of the genetic diversity in the *Chenopodium* accessions. These traits can be considered important distinguishing traits for studying genetic diversity in *Chenopodium. Chenopodium* is known for its grain content, but it also has good fodder‐yielding qualities; the accessions IC109734 and IC415477 of *Chenopodium album* are suitable as a good fodder crop. Earlier, quinoa breeders utilized the attributes such as plant height, inflorescence diameter, stem diameter, and seed yield and concluded that all the traits were strongly interlinked with each other (Al‐Naggar et al., 2017). A previous report also suggested a negative correlation between panicle biomass and reproductive efficiencies in *Chenopodium quinoa* (Bertero & Ruiz, 2010).

Here, we have also conducted a proximate composition analysis of *Chenopodium* accessions. The maximum ash content of 3.24% was observed in accession EC507744. Similar results for ash content of 3.4% in Quinoa were reported in a study by (Cardozo et al., 1979). High content of protein in *C. quinoa* seeds (5.5 g/100 g) followed by *C. album* (3.4 g/100 g) was also reported by (Pachauri et al., 2017). Previous studies have also reported the high protein content in quinoa seeds (12%–19%) (Ahamed et al., 1998). The studies conducted by many authors reported Quinoa as an excellent source of dietary fiber. So high dietary fiber accessions EC507741 and IC415477 reported in this study can be taken up by breeders for further studies (Fardet, 2010; González Martín et al., 2014; Jancurová et al., 2009; Lamothe et al., 2015; Saturni et al., 2010). We reported a high concentration of sugar in accessions IC411824 and IC411825. Similarly, Ogungbenle (2003) evaluated the sugar content and chemical composition of seed flour of Quinoa and reported a high proportion of d‐xylose (120 mg/100 g) and maltose (101 mg/100 g), and a low level of glucose (19 mg/100 g) and fructose (19.6 mg/100 g) (Ogungbenle, 2003). Thus, quinoa could be effectively utilized in the beverage industry to prepare malted drink formulations. Earlier studies reported the content of starch in quinoa ranged from 51% to 60% of grain weight (Atwell et al., 1983; Bastidas et al., 2016; Koziol, 1992). It is in concurrence with our study; accession IC411825 was reported to have the highest starch content (45.3%). Starch obtained from quinoa can be used for specialized industrial applications due to its small granules and high viscosity (Galwey et al., 1990). Here, we reported a high amount of mineral contents such as Cu, Fe, Zn, and Mg in *C. quinoa* accessions compared to *C. album*, which agreed with the previous studies. A previous study reported a significantly high level of iron and zinc in *C. Quinoa* than in *C. album* seeds (Pachauri et al., 2017). In a similar study, high content of iron (948.5 mg/kg), phosphorus (2735–4543.3 mg/kg), potassium (9562.2 mg/kg), and magnesium (1901.5 mg/kg) has been observed in quinoa (González Martín et al., 2014; Jancurová et al., 2009; Nascimento et al., 2014). Similarly, quinoa's higher calcium content (874 mg/kg) has also been reported (Jancurová et al., 2009; Sharma et al., 2012). A study reported that in seeds of sweet genotypes of *C. quinoa*, saponin content varies from0.02% to 0.04% dry matter, and in bitter genotypes, saponin content varies from 0.47% to 1.13% (Mastebroek et al., 2000). So, it may be concluded that saponin content in quinoa may be responsible for the sweet or bitter taste of quinoa genotypes, and can be used as a marker to separate sweet and bitter genotypes. Saponins have immense industrial importance and are used in preparing soaps, detergents, shampoos, beer, fire extinguishers, photography, and cosmetic and pharmaceutical industries (Johnson et al., 1993). So, accessions with high saponin content may be useful as an industrial source. Studies at the molecular level in *Chenopodium* species would further help dissect pathways related to differential traits in Chenopodium, which would be further utilized by breeders and researchers (Salazar et al., 2019).

Our study revealed the presence of a high amount of essential amino acids in *Chenopodium* accessions. Other studies have also reported higher content of essential amino acids in Quinoa as compared to common cereals (Ruales & Nair, 1992; Wright et al., 2002). Poonia and Upadhayay (2015) also reported amino acid content in quinoa seeds was high (57.7%) except for lysine and leucine. Similar results were obtained in our study, in which *C. quinoa* accessions had higher amounts of essential amino acids than *C. album*. Generally, the protein content of quinoa ranges from ~13% to 16.5% and it contains 10 essential amino acids. It has a high content of amino acids, lysine, and methionine, which are deficient in many grains. It is considered as best vegetable protein source similar to those in milk, and even higher than those present in cereal grains such as rice, wheat, and maize. Quinoa can fulfill amino acid requirements in adults: 180% of histidine (his), 338% of lysine (lys), 274% of isoleucine (ile), 320% of phenylalanine+tyrosine (phe + tyr), 212% of methionine+cysteine (met+cys), 331% of threonine (thr), 323% of valine (val), and 228% of tryptophan (trp) (Bastidas et al., 2016). It is also considered a valuable source of nutrition, especially for infants (Abugoch et al., 2008). Such genetic diversity might be due to factors like the genetic architecture of population, heterogeneity, history of selection, and/or developmental traits and has been reported in different crop species (Alemayehu & Becker, 2002; Ghafoor et al., 2001; Singh, 1991; Singh et al., 2004). Quinoa seeds are a good source of seed protein and have appreciable amount of seed carotenoids, which is in agreement with earlier reports on this plant (Cardozo et al., 1979; Wright et al., 2002). A recent study identified 60 genes (agamous‐like 7, far‐red impaired response 1, ethylene‐responsive element‐binding factor 5, sh4‐related 3, a basic helix‐loop‐helix (bHLH) DNA‐binding protein, etc.) having functional roles in protein synthesis, sugar transport, starch transport, and embryo and seed size development in quinoa cultivars Titicaca, Regalon Baer, and Pasankalla rosada. They suggested high‐precision candidate markers for nutritional traits in quinoa (Grimberg et al., 2022). Previous studies also showed the value of Chenopodium leaves as a vegetable supplement; such studies might be considered in *C. album* for future studies (Bhargava et al., 2019). Moreover, transcriptomics and metabolomics approaches would further provide a deep understanding of molecular mechanisms revealing the tolerant nature of crop Chenopodium and the pathway involved in metabolites related to nutritional factors (Huan et al., 2022). The present study shows that *C. album* had low seed yield and small, light weighted seeds with high protein content. We carried out a comparative study of *C. quinoa* and *C. album* based on agro‐morphological, nutritional traits and discussed that accession of neglected crop *C. album* had more nutritional value than *C. quinoa*. Therefore, a selective breeding program would further help in achieving the desired gain in Chenopodium and related species to fulfill the increasing demand for food supply in upcoming years globally. To the best of our knowledge, this study is the first report in which both agro‐morphological as well nutritional traits are taken into account to compare two different Chenopodium species. We discovered a more nutritive *C. album* accession, IC415477, as compared to other accessions of *C. quinoa*.

## 5 CONCLUSIONS

The results of the study imply that seeds of *C. album* are at par with the *C. quinoa* seeds and well‐endowed with essential nutrients required for human consumption. The *C. album* accession IC415477 was nutritionally at par or higher for test weight, and nutritional parameters with *C. quinoa* accessions and seed yield per plant for IC415477 (117.02 g) was ~1.88 times more than the highest observed in *C. quinoa* group (EC507742—62.16 g). IC415477 can be promoted in the farmer's community for cultivation to meet sustainable development goals (SDGs, Goal 1: no poverty; Goal 2: zero hunger), uplifting poor farmer's income and livelihood, and worldwide food security. Being a native species, IC415477 would have higher acceptability and adaptability among general people as compared to *C. quinoa*. To the best of our knowledge, this is the first study analyzing two different species in the genus *Chenopodium*. Based on nutritional profiling and phenotypic studies, we found accession IC415477 of *C. album* to be the best candidate accession which can be utilized by breeders/researchers for a future breeding program to raise more nutritive Chenopodium crop.

## AUTHOR CONTRIBUTIONS

**Rakesh Bhardwaj:** Conceptualization (lead); data curation (equal); formal analysis (equal); investigation (lead); methodology (equal); project administration (equal); supervision (equal); validation (equal); visualization (equal); writing – original draft (equal); writing – review and editing (equal). **Rashmi Yadav:** Conceptualization (lead); data curation (equal); formal analysis (equal); investigation (equal); methodology (lead); project administration (lead); supervision (equal); validation (equal); visualization (equal); writing – original draft (equal); writing – review and editing (equal). **Harinder Vishwakarma:** Conceptualization (supporting); data curation (lead); formal analysis (lead); investigation (supporting); visualization (lead); writing – original draft (supporting); writing – review and editing (supporting). **Kriti Sharma:** Validation (supporting); visualization (supporting); writing – original draft (lead); writing – review and editing (equal). **Rahul Chandora:** Resources (lead); writing – review and editing (supporting). **Jai Rana:** Conceptualization (lead); funding acquisition (lead); project administration (lead); resources (equal); supervision (equal); validation (equal); writing – review and editing (equal). **Amritbir Riar:** Conceptualization (supporting); funding acquisition (lead); project administration (lead); validation (equal); writing – review and editing (equal).

## CONFLICT OF INTEREST STATEMENT

The authors declare that they have no competing financial interests and personal relationships that could have appeared to influence the work reported in this paper.

## Supporting information


Table S1.

## Data Availability

All data generated or analyzed during this study are included in this article.
