# Supplementary material for: Agro‐morphological and nutritional assessment of chenopod and quinoa germplasm—Highly adaptable potential crops
Source: Food Sci Nutr. 2023 Jul 12;11(9):5446–59. doi: 10.1002/fsn3.3502 (PMC10494622; doi:10.1002/fsn3.3502)
Supplement: Supplementary file 1 — Table S1. [file FSN3-11-5446-s001.docx]

**Table S1 Pearson’s correlation coefficient between agro-morphological and nutritional traits of Chenopodium**

| **Traits** | **FF** | **IL** | **LL** | **LW** | **PH** | **Mat** | **YPP** | **TW** | **Mo** | **Ash** | **Pro** | **DF** | **Oil** | **Su** | **St** | **Ph** | **Sp** | **Iron** | **Fe** | **Zn** | **Mg** |
| --- | --- | --- | --- | --- | --- | --- | --- | --- | --- | --- | --- | --- | --- | --- | --- | --- | --- | --- | --- | --- | --- |
| **FF** | 1 | 0.629 | 0.462 | 0.57 | .892^**^ | .721^*^ | 0.505 | -0.572 | -0.207 | 0.222 | 0.055 | .676^*^ | .698^*^ | -.897^**^ | -.717^*^ | .680^*^ | 0.335 | -0.631 | -0.516 | -.858^**^ | -0.151 |
| **IL** |  | 1 | 0.463 | 0.253 | 0.589 | 0.578 | 0.455 | -.687^*^ | -0.481 | -0.231 | -0.42 | 0.472 | .864^**^ | -.695^*^ | -0.421 | .777^**^ | 0.596 | -.633^*^ | -0.301 | -0.48 | -0.005 |
| **LL** |  |  | 1 | .917^**^ | .743^*^ | 0.455 | .874^**^ | -0.061 | -0.125 | -0.32 | -0.27 | .699^*^ | 0.585 | -0.471 | -.633^*^ | 0.598 | 0.372 | -.698^*^ | -.772^**^ | -0.219 | -0.597 |
| **LW** |  |  |  | 1 | .802^**^ | 0.508 | .794^**^ | -0.058 | -0.004 | -0.16 | -0.038 | .638^*^ | 0.473 | -0.479 | -0.617 | 0.428 | 0.203 | -.668^*^ | -.771^**^ | -0.34 | -.688^*^ |
| **PH** |  |  |  |  | 1 | .752^*^ | .651^*^ | -0.438 | -0.038 | 0.065 | -0.156 | .756^*^ | .634^*^ | -.764^*^ | -.752^*^ | .689^*^ | 0.354 | -.723^*^ | -.779^**^ | -.720^*^ | -0.316 |
| **Mat** |  |  |  |  |  | 1 | 0.334 | -0.565 | 0.023 | -0.321 | -0.119 | 0.409 | 0.529 | -.634^*^ | -0.433 | 0.456 | 0.205 | -0.524 | -0.499 | -0.536 | -0.135 |
| **YPP** |  |  |  |  |  |  | 1 | 0.097 | -0.184 | -0.264 | -0.018 | .813^**^ | 0.592 | -.667^*^ | -.772^**^ | .755^*^ | 0.236 | -.704^*^ | -.763^*^ | -0.239 | -0.584 |
| **TW** |  |  |  |  |  |  |  | 1 | 0.48 | -0.088 | 0.357 | -0.002 | -.656^*^ | 0.347 | -0.015 | -0.194 | -0.462 | 0.388 | -0.079 | 0.496 | -0.163 |
| **Mo** |  |  |  |  |  |  |  |  | 1 | -0.026 | 0.394 | -0.113 | -.741^*^ | 0.214 | 0.093 | -0.213 | -.743^*^ | 0.372 | -0.221 | 0.231 | -0.194 |
| **Ash** |  |  |  |  |  |  |  |  |  | 1 | 0.209 | 0.145 | -0.154 | -0.004 | -0.179 | -0.077 | -0.006 | 0.299 | 0.178 | -0.323 | 0.378 |
| **Pro** |  |  |  |  |  |  |  |  |  |  | 1 | -0.036 | -0.37 | -0.134 | -0.041 | -0.168 | -.750^*^ | 0.409 | 0.22 | 0.131 | -0.323 |
| **DF** |  |  |  |  |  |  |  |  |  |  |  | 1 | 0.526 | -.761^*^ | -.986^**^ | .859^**^ | 0.33 | -0.489 | -.731^*^ | -0.489 | -0.126 |
| **Oil** |  |  |  |  |  |  |  |  |  |  |  |  | 1 | -.703^*^ | -0.498 | .675^*^ | .728^*^ | -.764^*^ | -0.312 | -0.555 | -0.136 |
| **Su** |  |  |  |  |  |  |  |  |  |  |  |  |  | 1 | .798^**^ | -.865^**^ | -0.283 | .635^*^ | 0.541 | .735^*^ | 0.179 |
| **St** |  |  |  |  |  |  |  |  |  |  |  |  |  |  | 1 | -.835^**^ | -0.311 | 0.468 | .708^*^ | 0.569 | 0.092 |
| **Ph** |  |  |  |  |  |  |  |  |  |  |  |  |  |  |  | 1 | 0.423 | -0.614 | -.637^*^ | -0.537 | -0.081 |
| **Sp** |  |  |  |  |  |  |  |  |  |  |  |  |  |  |  |  | 1 | -0.516 | -0.155 | -0.512 | 0.312 |
| **Iron** |  |  |  |  |  |  |  |  |  |  |  |  |  |  |  |  |  | 1 | .713^*^ | 0.571 | 0.42 |
| **Fe** |  |  |  |  |  |  |  |  |  |  |  |  |  |  |  |  |  |  | 1 | 0.407 | 0.41 |
| **Zn** |  |  |  |  |  |  |  |  |  |  |  |  |  |  |  |  |  |  |  | 1 | -0.136 |
| **Mg** |  |  |  |  |  |  |  |  |  |  |  |  |  |  |  |  |  |  |  |  | 1 |

**Correlation is significant at the 0.01 level (2-tailed), *Correlation is significant at the 0.05 level (2-tailed). **Abbreviations** FF: %50 Flowering, IL: Inflorescence length, LL: Leaf length, LW: Leaf width, PH: Plant height, Mat: 80% Maturity, YPP: Seed yield per plant, TW: Test weight, Mo: Moisture content, Ash: Ash content, Pro: Protein content, DF: Dietary fibre, Oil: Oil content, Su: Sugar content, St: Starch content, Ph: Phenol content, Sp: Saponin content, Cu: Copper, Fe: Iron, Zn: Zinc, Mg: Magnesium.
